# Sexually transmitted infections among female sex workers tested at STI clinics in the Netherlands, 2006–2013

**DOI:** 10.1186/s12982-015-0034-7

**Published:** 2015-08-28

**Authors:** Maud M. A. Verscheijden, Petra J. Woestenberg, Hannelore M. Götz, Maaike G. van Veen, Femke D. H. Koedijk, Birgit H. B. van Benthem

**Affiliations:** Epidemiology and Surveillance Unit, Centre for Infectious Diseases Control, National Institute for Public Health and the Environment (RIVM), Bilthoven, The Netherlands; Radboud University, Nijmegen, The Netherlands; Department of Infectious Disease Control, Municipal Public Health Service Rotterdam-Rijnmond, Rotterdam, The Netherlands; STI Outpatient Clinic, Public Health Service of Amsterdam, Amsterdam, The Netherlands; Municipal Health Service Twente, Enschede, The Netherlands

**Keywords:** Sexually transmitted infections (STI), HIV, STD, Female sex workers, Commercial sex

## Abstract

**Background:**

Specialised sexually transmitted infection (STI) clinics in the Netherlands provide STI care for high-risk groups, including female sex workers (FSW), at the clinic and by outreach visiting commercial sex workplaces with a permit. The objective was to investigate the STI positivity rate and determinants of an STI diagnosis among FSW tested by STI clinics in the Netherlands.

**Methods:**

Sexually transmitted infection clinics report demographic, behavioural and diagnostic information of every consultation to the National Institute for Public Health and the Environment. We analysed all consultations of FSW between 2006 and 2013. Trends in STI positivity rate (chlamydia, gonorrhoea, infectious syphilis, HIV and hepatitis B) were analysed using χ^2^ for trend and logistic regression was used to analyse determinants associated with an STI diagnosis. Differences between consultations at the STI clinic and consultations during outreach were analysed using χ^2^ tests.

**Results:**

The positivity rate for any STI (overall 9.5 %) was stable from 2006 to 2013. Chlamydia positivity rate (overall 7.1 %) decreased (p < 0.001) and gonorrhoea positivity rate (overall 2.6 %) increased (p < 0.001). For gonorrhoea, the highest positivity rate was found oropharyngeal (2.0 %). Characteristics associated with STI were a younger age [adjusted odds ratio (aOR) 0.96, 95 % confidence interval (CI) 0.95–0.97 per year], a previous STI diagnosis (aOR 1.63, 95 % CI 1.38–1.92) and being notified for an STI by partner notification (aOR 2.61, 95 % CI 2.0–3.40). The STI positivity rate was significantly lower among FSW tested at outreach locations (8.6 %) compared to FSW tested at the STI clinic (11.7 %, p < 0.001).

**Conclusions:**

The STI positivity rate among FSW remained stable, but underlying this was a decreasing chlamydia trend and an increasing gonorrhoea trend, suggesting a shift in STI risks among FSW over time. Condom use during oral sex should be promoted since oropharyngeal gonorrhoea was frequently diagnosed and because of the potential spread of antimicrobial resistant gonococci.

**Electronic supplementary material:**

The online version of this article (doi:10.1186/s12982-015-0034-7) contains supplementary material, which is available to authorized users.

## Background

The policy concerning commercial sex work in the Netherlands is quite different compared to other countries. In many countries, selling of sex is illegal (Belgium [1], South-Africa [2], Canada [3] and most states in the United States of America [4]) or the purchase of sex is considered a legal offence (Sweden [5]). In the Netherlands, commercial sex work is legal since the brothel prohibition was lifted in 2000 [6]. In order to sell sex, employers must obtain a permit from the municipalities. The goal of the legalisation was to regulate voluntary prostitution, to decrease the amount of involuntary prostitution and to improve the social position of sex workers. However, the legislation may have led to an increase in illegal prostitution and some argue that the quality of work circumstances of sex workers (legal and illegal) did not improve [7].

Female sex workers (FSW) are considered a high-risk group for acquisition of sexually transmitted infection (STI) [8, 9], due to their social vulnerability and factors associated with their work like a history of multiple sex partners, inconsistent condom use or co-infection with other STI [10, 11]. Even though in the Netherlands there were lower STI rates among FSW compared to other high-risk groups [12], FSW remain a high-risk group for STI transmission to their numerous clients. There is a potential for some of these clients to act as a bridging population for further spread of STI into the general population [13–15]. Because FSW are at high-risk for STI acquisition and transmission, they are one of the target groups for free STI care at specialised STI clinics located at public health services throughout the Netherlands. These STI clinics provide STI care additional to the regular national health services (like general practitioners), to reach people who might otherwise not seek STI care timely [16]. The STI clinics are funded by the government based on the subsidy regulation Sexual Health [17]. High-risk groups, including FSW, can visit these clinics for STI testing. Some STI clinics also perform outreach activities for FSW, where they routinely visit known commercial sex workplaces such as brothels, sex clubs and window-based prostitution in order to provide STI counselling, testing and hepatitis B vaccination. There is wide variation between STI clinics in the frequency they perform outreach activities, varying from weekly to yearly. All workplaces with a permit for commercial sex work are obligated to provide access to staff of the STI clinic and public health services [18]. Although testing is voluntarily, FSW are advised to go for STI testing four times a year [19].

Although FSW are an important population for STI prevention and control, information about the prevalence and trends of STI among FSW is limited in the Netherlands, compared to other risk groups like young people and men having sex with men [20]. In addition, little is known about the associated determinants for different STI. More insight into the characteristics of this population and the determinants associated with STI occurrence might help target STI control. Therefore, the objective of our study was to investigate the STI positivity rate, trends in STI and associated determinants among FSW who received STI care by the STI clinics (through a visit or by outreach) between 2006 and 2013 in the Netherlands. Secondary aim was to investigate differences between FSW tested through a visit or by outreach.

## Methods

For this study, we used surveillance data from STI clinics who all routinely submit an anonymised predefined set of variables (demographics, sexual behaviour, and diagnostic information) of the medical records of each consultation to the Dutch National Institute of Public Health and the Environment (RIVM). Data of the consultations are extracted from the medical records and transferred to the RIVM using a secured web-based application named SOAP [12].

### Study population

We included all consultations reported by all STI clinics in the Netherlands between 2006 and 2013 of FSW of 18 years or older, the legal age to perform sex work in the Netherlands. A FSW was defined as a woman who self-reported to have exchanged sex (vaginal, anal or oral) for money or other valuable goods in the 6 months prior to the consultation at an STI clinic. During every consultation, the staff of the STI clinic asks whether the visitor had exchanged sex for money or other valuable goods. Due to the anonymous character of the available data, we cannot retrieve whether the data contains repeated consultations of an individual FSW. Therefore, the unit of analysis is a consultation.

### Data

Variables included in this study were age; year of consultation; ethnicity; previous STI diagnosis (chlamydia, gonorrhoea or syphilis) in the last 2 years; ever HIV tested; STI symptoms; current STI diagnosis (chlamydia, gonorrhoea, syphilis, HIV, hepatitis B); whether the FSW was notified for an STI through partner notification and condom use at last sexual contact and whether this was a casual (paying or non-paying) or steady partner. Except for current STI diagnoses, all variables were self-reported. The categories of the variables are presented in Table 1. Due to a policy chance in the registration, ethnicity was a combination variable that consisted of self-defined ethnicity (from 2006 until 2010) and ethnicity based on (parental) country of birth (from 2011 until 2013) [21]. The variables STI symptoms and partner notification were reported since 2007 and the variable condom use at last sexual contact, and whether this contact was a steady or a casual partner were reported since 2011. Degree of urbanisation of the attendees’ residence (five categories based on population density of the postal code area, see Table 1), was added to the database and analysed for the years 2006–2013 [22]. Because we used data that was routinely collected for surveillance purposes and data was obtained anonymously, no ethical approval was needed.

**Table 1:** Characteristics of female sex workers tested by STI clinics in the Netherlands, 2006–2013

	2006	2007	2008	2009	2010	2011	2012	2013	Total
	%	%	%	%	%	%	%	%	%
Number of consultations (n)	2854	3367	3810	4214	4971	5301	5877	5770	36,164
Age (median)	29	28	29	30	30	30	30	31	30
Ethnicity^a^									
Native Dutch	51.7	45.1	43.9	44.3	42.6	25.8	29.6	31.6	37.6
Turkish	0.2	0.2	0.3	0.3	0.2	0.3	0.3	0.3	0.3
North-African	1.1	1.2	0.8	0.5	0.5	2.5	2.2	2.3	1.5
Surinamese	1.4	1.3	1.1	1.0	0.6	2.9	3.7	3.6	2.2
Dutch Antillean	0.7	0.4	0.5	0.2	0.3	1.3	1.8	1.8	1.0
East-European	15.3	16.4	17.5	21.5	23.2	34.4	33.0	31.0	25.6
Sub-Saharan African	2.3	2.0	1.8	1.8	2.0	2.6	2.8	2.9	2.3
Mid-South American	8.5	8.0	9.2	9.3	9.1	10.1	11.8	11.6	10.0
Other European	13.7	19.5	19.7	16.4	16.6	4.8	5.0	4.9	11.5
Asian	2.3	2.4	2.9	2.9	3.5	5.8	5.2	6.4	4.3
Unknown	0.2	0.2	0.3	0.2	0.1	9.5	4.5	3.4	2.8
Rest	2.5	3.2	2.1	1.7	1.4	0.1	0.1	0.2	1.2
Ever HIV tested									
No	27.5	20.0	16.6	14.4	14.3	12.6	10.7	9.3	14.5
Yes, positive	0.1	0.2	0.1	0.1	0.1	0.2	0.2	0.3	0.2
Yes, negative	63.6	72.6	76.1	79.3	80.9	81.7	83.3	85.5	79.3
Unknown	8.8	7.2	7.2	6.2	4.7	5.5	5.8	4.9	6.1
STI in last 2 years^b^									
No	50.2	55.2	82.9	81.2	82.0	84.2	83.4	81.8	77.5
Yes	18.3	13.5	11.1	10.5	10.3	7.6	8.4	8.8	10.4
Unknown	31.6	31.3	6.0	8.3	7.6	8.2	8.2	9.4	12.0
Symptoms^c^									
No		23.8	71.2	76.5	77.5	76.0	79.6	82.5	66.5
Yes		15.9	25.2	21.6	21.7	23.5	19.7	17.0	19.0
Unknown		60.3	3.6	1.9	0.8	0.5	0.7	0.5	15.4
Notified for an STI by partner notification^c^									
No		31.6	94.5	97.9	97.9	97.7	96.2	96.5	83.1
Yes		0.7	1.9	1.3	1.4	2.0	2.1	2.2	1.6
Unknown		67.7	3.6	0.8	0.6	0.3	1.7	1.3	14.5
Degree of urbanisation									
Very high	33.6	37.5	38.8	36.7	32.4	31.2	29.8	29.7	33.1
High	15.0	14.6	16.7	15.7	14.4	14.5	13.9	13.9	14.7
Medium	8.4	7.8	8.6	8.5	7.4	6.3	7.1	7.9	7.6
Low	3.0	3.6	4.3	4.2	4.1	4.0	4.6	4.0	4.1
Very low	3.3	2.8	3.4	4.1	4.1	3.8	3.9	3.2	3.6
Unknown	36.6	33.7	28.1	30.7	37.5	40.1	40.7	41.2	36.8
Condom use at last sexual contact by type of partner^c^									
No condom use with steady partner						29.1	15.7	16.8	20.3
Yes condom use with steady partner						2.8	5.2	2.6	3.6
No condom use with casual partner						7.6	10.8	9.2	9.2
Yes condom use with casual partner						51.6	60.4	64.1	58.9
Unknown						8.9	8.0	7.3	8.1

*STI* sexually transmitted infections

^a^Ethnicity was based on self-defined ethnicity for 2006–2010 and on (parental) country of birth from 2011–2013

^b^Diagnosed with chlamydia, gonorrhoea or syphilis in the last 2 years

^c^Not reported for all years

Each STI consultation involved laboratory testing and, if indicated, medical examination by a physician or specialised nurse. FSW were offered standard testing for chlamydia, gonorrhoea, syphilis and HIV (nation-wide opt-out policy since 2007, meaning that FSW are standard tested for HIV unless they object [23, 24]). As FSW are indicated for vaccination against hepatitis B, they are screened for hepatitis B. Chlamydia and gonorrhoea were tested vaginally, oropharyngeal and, if indicated, anorectal. All laboratories used nucleic acid amplified tests (NAAT) to diagnose chlamydia and gonorrhoea. Culture of *Neisseria Gonorrhoeae* was sometimes performed in symptomatic cases or in positive NAAT tests. Syphilis screening was done using *Treponema pallidum* haemagglutination or *Treponema pallidum* particle agglutination (TPHA/TPPA) tests followed by a venereal disease research laboratory (VDRL) test and/or a fluorescent treponemal antibody (FTA) test. Infectious syphilis included diagnoses of primary and secondary syphilis as well as Lues latens recens. HIV was tested by enzyme-linked immunosorbent assay (ELISA) combotest (antibody/p24 antigen) confirmed by Western Blot test. Hepatitis B core antibody was used as screening-test for hepatitis B, and if positive hepatitis B surface antigen and anti-HBs were tested [25].

### Outcome measures and statistical analyses

We calculated the STI positivity rate as the number of consultations in which at least one STI (chlamydia, gonorrhoea, infectious syphilis, HIV, hepatitis B) was diagnosed per 100 consultations. STI specific positivity rates were calculated out of those consultations where a laboratory test was performed for these specific STI. To increase the readability, we will report the STI positivity rate among FSW as a percentage when we mean the STI positivity rate per 100 consultations. χ^2^ test for trend was performed to assess trends in STI positivity rates over time. Positivity rates by anatomic location were calculated for the years 2008–2013.

Uni- and multivariable logistic regression analyses for any STI, chlamydia and gonorrhoea were done for the years 2011–2013, because this represents the most recent situation and this avoids the break in trend concerning the definition of ethnicity. Since it is an explorative study, all variables described above were included in the univariable analysis as well as in the multivariable analysis. For the outcomes infectious syphilis, HIV and hepatitis B, univariable analyses were done for the years 2006–2013, due to the small amount of positive diagnoses.

Most STI clinics (22 of 26) performed outreach activities. Six of those clinics used different coding for the consultations at the clinic and outreach consultations. In order to investigate whether there were differences between FSW tested at the STI clinic and FSW tested during outreach, we analysed the characteristics and STI positivity rate separately according to location of testing using a χ^2^ test. All analyses were done using SPSS version 18 with a significance level of p < 0.05.

## Results

### Characteristics of study population

Between 2006 and 2013, 801,864 consultations were registered at the STI clinics of which 36,296 (4.5 %) were among FSW. Of all consultations among FSW 36,164 (99.6 %) were 18 years or older. The number of consultations among FSW aged 18 years or older increased from 2854 (4.1 % of all consultations) in 2006 to 5877 (4.8 % of all consultations) in 2012. In 2013, there were 5770 consultations among FSW (4.3 % of all consultations).

The median age of all FSW was 30 years (interquartile range 24–38 years). In the period 2006–2010 using self-defined ethnicity, the greatest number of consultations were among native Dutch FSW (45.0 %), while in the period 2011–2013 using (parental) country of birth, the greatest number of consultations were among East-European FSW (32.8 %). The percentage of FSW ever tested for HIV increased from 63.7 % in 2006 to 85.8 % in 2013. Of the consultations in 2011–2013 where the last sexual contact was a steady partner, a condom was used in 15.1 %, while a condom was used in 58.9 % of the consultations where the last sexual contact was a casual (paying or non-paying) partner (Table 1).

### STI positivity rate

**Fig. 1:**
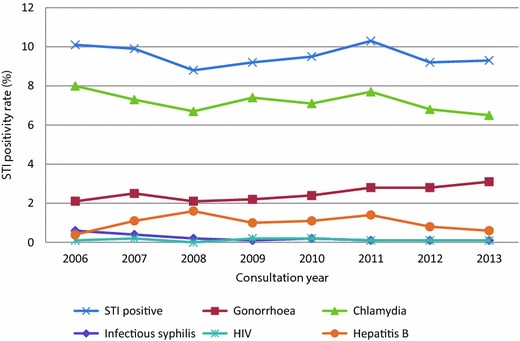
STI positivity rate among FSW tested by STI clinics in the Netherlands, 2006–2013

The overall STI positivity rate among FSW was 9.5 %, and was stable throughout the study period (χ^2^ for trend, p = 0.5). The positivity rate for chlamydia decreased over the years from 8.0 % in 2006 to 6.5 % in 2013 (χ^2^ for trend, p = 0.03). For gonorrhoea, the positivity rate increased from 2.1 % in 2006 to 3.1 % in 2013 (χ^2^ for trend, p < 0.001). The positivity rate for infectious syphilis decreased from 0.6 % in 2006 to 0.1 % in 2013 (χ^2^ for trend, p < 0.001). Positivity rates for HIV and hepatitis B remained stable [overall 0.1 % (p = 0.27) and 1.0 % (p = 0.12) respectively] (Fig. 1).

Table 2 shows the positivity rate by anatomic location for chlamydia and gonorrhoea. For chlamydia, the highest positivity rate was found anorectal (6.2 %), while for gonorrhoea the highest positivity rate was found oropharyngeal (2.0 %). Of the 780 gonorrhoea diagnoses between 2008 and 2013, 418 (53.6 %) were diagnosed in the oropharynx. This percentage increased from 35.8 % in 2008 to 59.0 % in 2013. There was also an increase in the percentage of consultations where gonorrhoea was tested oropharyngeal; of the 3810 consultations in 2008, there were 1870 consultations (49.1 %) where gonorrhoea was tested oropharyngeal, in 2013 this was in 4725 of the 5770 consultations (81.9 %).

**Table 2:** Number of tests and positivity rate for chlamydia and gonorrhoea by anatomic location

	2008	2009	2010	2011	2012	2013	Total
Number of consultations	3810	4214	4971	5301	5877	5770	29,943
Chlamydia diagnoses^a^	254	311	352	408	397	373	2095
Chlamydia urogenital							
n tested	3762	4177	4932	5287	5861	5751	29,770
n positive (% positive)	237 (6.30)	275 (6.58)	312 (6.33)	345 (6.53)	327 (5.58)	303 (5.27)	1799 (6.04)
Chlamydia oropharyngeal							
n tested	933	1264	1925	3761	4407	4511	16,801
n positive (% positive)	18 (1.93)	31 (2.45)	51 (2.65)	89 (2.37)	86 (1.95)	67 (1.49)	342 (2.04)
Chlamydia anorectal							
n tested	515	599	697	1163	1754	2343	7071
n positive (% positive)	38 (7.38)	47 (7.85)	42 (6.03)	80 (6.88)	101 (5.76)	129 (5.51)	437 (6.18)
Gonorrhoea diagnoses^a^	81	93	120	146	162	178	780
Gonorrhoea urogenital							
n tested	3762	4173	4933	5286	5805	5749	29,708
n positive (% positive)	57 (1.52)	55 (1.32)	84 (1.70)	94 (1.78)	104 (1.79)	103 (1.79)	497 (1.67)
Gonorrhoea oropharyngeal							
n tested	1870	2339	3305	3869	4519	4725	20,627
n positive (% positive)	29 (1.55)	37 (1.59)	70 (2.12)	84 (2.17)	93 (2.06)	105 (2.22)	418 (2.03)
Gonorrhoea anorectal							
n tested	1134	1345	1657	2124	2658	2822	11,740
n positive (% positive)	22 (1.94)	19 (1.41)	18 (1.09)	27 (1.27)	30 (1.13)	29 (1.03)	145 (1.24)

^a^All infections diagnosed, including those based on pooled samples and infection with an unknown anatomic location. Infections do not add to the total number of diagnoses since infection can occur in multiple locations

### Determinants of STI

The multivariable analysis showed that a younger age, previous STI diagnosis, and being notified for an STI were significantly associated with an STI (chlamydia, gonorrhoea, infectious syphilis, HIV and/or hepatitis B), chlamydia and gonorrhoea diagnoses (Table 3). FSW previously tested HIV negative were significantly less likely to be diagnosed with chlamydia compared to FSW who were never HIV tested [adjusted odds ratio (aOR) 0.45, 95 % confidence interval (CI) 0.39–0.53]. An East-European ethnicity was associated with a higher risk for gonorrhoea compared to native Dutch (aOR 1.44, 95 % CI 1.19–1.84), but with a lower risk for chlamydia (aOR 0.79, 95 % CI 0.67–0.93). FSW with a Sub-Saharan African, a Mid-South American or a North-African ethnicity were also less likely to be diagnosed with chlamydia than native Dutch FSW (Table 3).

**Table 3:** Uni- and multivariable logistic regression for determinants of STI among FSW, 2011–2013

	Any STI^a^, N = 16,948			Chlamydia, N = 16,933^b^			Gonorrhoea, N = 16,932^b^		
	N positive	Crude OR (95 % CI)	aOR (95 % CI)	N positive	Crude OR (95 % CI)	aOR (95 % CI)	N positive	Crude OR (95 % CI)	aOR (95 % CI)
Age	1624	*0.95* (*0.95–0.96*)	*0.96* (*0.95–0.97*)	1178	*0.95* (*0.94–0.95*)	*0.95* (*0.95*–*0.96*)	486	*0.95* (*0.94*–*0.96*)	*0.97* (*0.96–0.98*)
Consultation year	1624	0.94 (0.88–1.0)	0.97 (0.91–1.04)	1178	*0.91* (*0.85–0.98*)	0.93 (0.86–1.01)	486	1.06 (0.95–1.19)	1.11 (0.99–1.25)
Ethnicity^c^									
Native Dutch	483	Reference	Reference	385	Reference	Reference	124	Reference	Reference
Turkish	5	0.90 (0.36–2.27)	0.66 (0.26–1.68)	4	0.91 (0.33–2.52)	0.61 (0.21–1.73)	2	1.43 (0.35–5.95)	1.37 (0.32–2.55)
North African	30	0.77 (0.52–1.13)	*0.59* (*0.40–0.87*)	22	0.71 (0.46–1.10)	*0.52* (*0.33–0.82*)	10	1.02 (0.53–1.97)	0.93 (0.48–1.80)
Surinamese	53	0.92 (0.68–1.24)	0.86 (0.63–1.16)	41	0.89 (0.64–1.24)	0.82 (0.58–1.15)	16	1.09 (0.64–1.85)	1.07 (0.63–1.84)
Dutch Antilleans	41	*1.61* (*1.14–2.28*)	1.30 (0.91–1.85)	31	*1.50* (*1.02–2.21*)	1.15 (0.77–1.72)	12	1.77 (0.97–3.24)	1.36 (0.73–2.54)
Eastern European	625	*1.17* (*1.03–1.32*)	1.01 (0.88–1.16)	427	0.98 (0.85–1.14)	*0.79* (*0.67*–*0.93*)	214	*1.55* (*1.24*–*1.95*)	*1.44* (*1.19*–*1.84*)
Sub-Saharan African	39	0.84 (0.60–1.19)	*0.65* (*0.46–0.92*)	13	*0.34* (*0.19–0.60*)	*0.25* (*0.14*–*0.44*)	9	0.76 (0.39–1.51)	0.78 (0.39–1.56)
Mid-South-American	128	*0.67* (*0.55–0.82*)	0.84 (0.68–1.04)	85	*0.56* (*0.44*–*0.71*)	*0.72* (*0.56*–*0.93*)	37	0.77 (0.53–1.12)	0.99 (0.67–1.46)
Other European	75	0.91 (0.71–1.18)	0.98 (0.76–1.28)	54	0.82 (0.61–1.10)	0.86 (0.64–1.17)	25	1.20 (0.78–1.86)	1.34 (0.86–2.09)
Asian	91	0.94 (0.74–1.19)	1.07 (0.84–1.37)	74	0.96 (0.74–1.25)	1.10 (0.84–1.44)	20	0.81 (0.50–1.30)	0.89 (0.55–1.45)
Unknown	91	*0.52* (*0.38*–*0.69*)	*0.57* (*0.42*–*0.77*)	40	*0.51* (*0.37*–*0.71*)	*0.58* (*0.41*–*0.82*)	16	0.65 (0.39–1.11)	0.77 (0.45–1.32)
Rest	3	1.38 (0.41–4.67)	0.98 (0.29–3.36)	2	1.12 (0.26–4.81)	0.80 (0.18–3.48)	1	1.76 (0.24–13.16)	1.24 (0.16–9.53)
STI in last 2 years									
No	1257	Reference	Reference	915	Reference	Reference	369	Reference	Reference
Yes	206	*1.75* (*1.49*–*2.05*)	*1.63* (*1.38*–*1.92*)	154	*1.77* (*1.48*–*2.12*)	*1.58* (*1.30*–*1.91*)	62	*1.71* (*1.30*–*2.25*)	*1.41* (*1.06*–*1.89*)
Unknown	161	*1.26* (*1.06*–*1.50*)	1.13 (0.91–1.39)	109	1.16 (0.95–1.43)	1.05 (0.82–1.35)	55	*1.46* (*1.09*–*1.94*)	1.11 (0.77–1.59)
Ever HIV tested									
No	325	Reference	Reference	256	Reference	Reference	79	Reference	Reference
Yes, positive	3	0.39 (0.12–1.26)	0.66 (0.20–2.21)	2	0.33 (0.08–1.39)	0.57 (0.13–2.48)	1	0.58 (0.08–4.30)	1.12 (0.15–8.48)
Yes, negative	1181	*0.42* (*0.37*–*0.48*)	*0.48* (*0.42*–*0.55*)	842	*0.39* (*0.34*–*0.45*)	*0.45* (*0.39*–*0.53*)	362	*0.58* (*0.45*–*0.75*)	0.77 (0.59–1.01)
Unknown	115	*0.67* (*0.53*–*0.84*)	0.80 (0.61–1.04)	78	*0.57* (*0.44*–*0.75*)	*0.71* (*0.52*–*0.96*)	44	1.12 (0.77–1.63)	1.36 (0.87–2.11)
Symptoms									
No	1174	Reference	Reference	854	Reference	Reference	341	Reference	Reference
Yes	437	0.55 (0.38–1.75)	1.24 (0.10–1.41)	312	*1.50* (*1.31*–*1.72*)	1.12 (0.97–1.30)	143	*1.70* (*1.39*–*2.07*)	*1.38* (*1.12*–*1.70*)
Unknown	13	1.64 (0.91–2.95)	1.37 (0.73–2.56)	12	*2.13* (*1.16*–*3.92*)	*2.01* (*1.04*–*3.88*)	2	0.83 (0.20–3.37)	0.44 (0.10–1.95)
Notified for an STI by partner notification									
No	1522	Reference	Reference	1105	Reference	Reference	450	Reference	Reference
Yes	83	*2.95* (*2.30*–*3.79*)	*2.61* (*2.0*–*3.40*)	61	*2.84* (*2.14*–*3.77*)	*2.19* (*1.61*–*3.00*)	29	*3.12* (*2.11*–*4.62*)	*2.59* (*1.70*–*3.93*)
Unknown	19	1.20 (0.68–1.77)	0.92 (0.54–1.54)	12	0.95 (0.53–1.71)	0.84 (0.44–1.61)	7	1.38 (0.64–2.95)	1.01 (0.44–2.30)
Degree of urbanisation									
Very high	485	Reference	Reference	344	Reference	Reference	154	Reference	Reference
High	220	0.97 (0.82–1.15)	1.06 (0.89–1.26)	166	1.04 (0.86–1.26)	1.09 (0.89–1.33)	61	0.85 (0.63–1.14)	0.99 (0.73–1.35)
Medium	113	0.99 (0.80–1.23)	1.10 (0.88–1.38)	85	1.05 (0.82–1.35)	1.12 (0.86–1.45)	31	0.85 (0.58–1.26)	1.02 (0.68–1.54)
Low	63	0.93 (0.71–1.22)	1.06 (0.80–1.41)	42	0.87 (0.63–1.21)	0.92 (0.65–1.30)	22	1.03 (0.65–1.62)	1.37 (0.86–2.19)
Very low	49	0.82 (0.60–1.12)	0.96 (0.70–1.31)	39	0.93 (0.66–1.31)	1.07 (0.75–1.52)	9	*0.48* (*0.24*–*0.94*)	0.61 (0.31–1.21)
Unknown	694	1.07 (0.95–1.21)	1.07 (0.94–1.21)	502	1.09 (0.95–1.26)	1.10 (0.95–1.28)	209	1.01 (0.82–1.25)	1.01 (0.81–1.27)
Gonorrhoea	486			121	*4.83* (*3.89*–*5.98*)	*3.77* (*3.01*–*4.72*)			
Chlamydia	1178						121	*4.83* (*3.89*–*5.98*)	*3.76* (*3.00*–*4.72*)
Condom use at last sexual contact by type of partner									
No condom use steady partner	368	Reference	Reference	279	Reference	Reference	95	Reference	Reference
Yes condom use steady partner	56	0.85 (0.64–1.15)	0.86 (0.64–1.17)	44	0.89 (0.64–1.24)	0.93 (0.66–1.30)	12	0.72 (0.39–1.31)	0.73 (0.40–1.35)
No condom use casual partner	200	*1.23* (*1.02*–*1.47*)	1.19 (0.98–1.44)	153	*1.23* (*1.00*–*1.51*)	1.21 (0.98–1.50)	53	1.24 (0.88–1.74)	1.14 (0.81–1.63)
Yes condom use casual partner	862	*0.79* (*0.69*–*0.90*)	0.89 (0.78–1.02)	611	*0.74* (*0.64*–*0.86*)	0.86 (0.73–1.00)	276	1.00 (0.79–1.27)	1.11 (0.86–1.42)
Unknown	138	0.94 (0.76–1.15)	1.06 (0.85–1.33)	91	0.81 (0.63–1.04)	0.91 (0.70–1.19)	50	1.34 (0.95–1.90)	*1.52* (*1.04*–*2.22*)

In italics: OR is statistically significant (p < 0.05)

*STI* sexually transmitted infections, *FSW* female sex worker, *OR* odds ratio, *aOR* adjusted odds ratio, *CI* confidence interval

^a^At least one positive diagnose for chlamydia, gonorrhoea, infectious syphilis, HIV or hepatitis B

^b^Only consultations were included where a related clinical test was done

^c^Ethnicity was based (parental) country of birth from 2011–2013

Infectious syphilis was significantly associated with a previous STI diagnosis and several ethnicities (such as North-African, Dutch Antilleans and East-European). HIV and hepatitis B were more frequently diagnosed among Sub-Saharan African and Mid-South American ethnicities than among native Dutch. Hepatitis B was significantly associated with STI related symptoms. A previous negative HIV test was associated with a lower risk for infectious syphilis, hepatitis B and HIV. Results can be found in Additional file 1.

### Outreach versus STI clinic

Table 4 shows the results of the comparison between outreach consultations and consultations performed at the STI clinics. The location of the consultation was known for 8606 consultations (24 % of all consultations) of which 34.2 % were outreach consultations. The STI positivity rate was significantly lower for outreach consultations compared to consultations at the STI clinics (8.6 and 11.7 % respectively, p < 0.001). FSW tested during outreach were older than FSW tested at the STI clinics (median age 32 versus 28 years, p < 0.001) and had more frequently a non-Dutch ethnicity (35.5 versus 30.9 %, p < 0.001).

**Table 4:** Characteristics and STI prevalence of FSW tested at STI clinic or tested during outreach activities

	STI clinic	Outreach	P-value
	%	%	
Number of consultations (n)	5560	2946	
Age (median)	28	32	<0.001
Ethnicity			
Native Dutch	35.5	30.9	<0.001
Turkish	0.4	0.0	0.002
North African	1.7	1.2	0.079
Surinamese	2.1	1.1	<0.001
Dutch Antillean	1.3	1.0	0.304
Eastern European	22.2	26.2	<0.001
Sub-Saharan African	2.5	1.8	0.028
Mid-South American	13.5	12.3	0.136
Other European	14.8	13.7	0.193
Asian	4.7	10.0	<0.001
Unknown	1.3	1.6	0.180
Rest	0.1	0.0	0.077
Previous STI			
No	72.6	80.5	<0.001
Yes	13.3	3.9	<0.001
Unknown	14.1	15.6	0.057
Ever HIV tested			
No	16.4	11.2	<0.001
Yes, positive	0.1	0.0	0.195
Yes, negative	72.3	74.5	0.027
Unknown	11.1	14.2	<0.001
Symptoms			
No	59.1	85.1	<0.001
Yes	22.8	12.4	<0.001
Unknown	18.1	2.5	<0.001
STI positivity rate			
STI overall	11.7	8.6	<0.001
Chlamydia	8.4	6.2	<0.001
Gonorrhoea	3.6	2.5	0.004
Infectious syphilis	0.4	0.1	0.009
HIV	0.2	0.1	0.078
Hepatitis B	1.6	1.5	0.877

Unknown for 27,558 consultations

*STI* sexually transmitted infections, *FSW* female sex worker

## Discussion

The number of consultations of FSW as well as the proportion FSW of all STI clinic consultations increased during the study period 2006–2013. The overall STI positivity rate among FSW who received care by the STI clinic remained stable, but there was a decrease in the chlamydia positivity rate and an increase in the gonorrhoea positivity rate. Determinants significantly associated with STI positivity were a younger age, a previous STI diagnosis and being notified by partner notification.

STI clinics in the Netherlands provide STI care to specific high-risk groups such as FSW, people below the age of 25 years and people with STI-related symptoms [16]. Compared to all female STI clinic attendees in 2013 [12], FSW were older, less often native Dutch, more often ever tested for HIV, more frequently using a condom in consultations where the last sexual contact was a casual partner and less often diagnosed with an STI (STI positivity rate of 9.5 % compared to 13.2 %) [12]. The chlamydia positivity rate among FSW in 2013 was relatively low compared to the chlamydia positivity rate among all women who attended these STI clinics (6.5 and 12.2 % respectively) [12]. The younger age, which appeared to be a risk factor for STI acquisition, and less frequent condom use with a casual partner among all female STI clinic attendees compared to FSW could explain this difference. Although among FSW the highest chlamydia positivity rate was found anorectal (5.5 % in 2013), this was still lower compared to all women who visited the STI clinic (10.2 % in 2013) [12]. The positivity rate of chlamydia that we found among FSW was comparable to the chlamydia prevalence among FSW in other West-European countries [26–29]. Countries outside Europe showed higher chlamydia prevalence [30, 31]. This could be explained by a lack of epidemiological surveillance systems and FSW-targeted health services in these countries leading to insufficient knowledge about FSW and their STI risk and how to target appropriate prevention. Indeed, there is a lower condom use with clients among FSW in countries outside Europe, compared to countries in Western Europe [30, 32]. The positivity rate of gonorrhoea in our study was comparable to the prevalence among FSW in other European countries (around 5 % or less [27, 28, 33]).

While the positivity rate for chlamydia was lower among FSW than among all women who attended the STI clinic, the positivity rate for gonorrhoea in our study was higher (3.1 % compared to 1.8 %) [12]. This may be explained by the high proportion of East-European FSW who have a higher gonorrhoea prevalence. Another explanation is the higher positivity rate for oropharyngeal gonorrhoea among FSW (2.2 % in 2013) than among all female STI clinic attendees (1.5 % in 2013), probably due to more unsafe oral sex contacts [12, 32]. FSW often do not use a condom when performing oral sex, because they can charge a higher fee for this service (personal communication with health care workers). We did find a relative high percentage of condom use in consultations where the last sexual contact was a casual partner, but perhaps this is reported for vaginal/anal intercourse only since oral sex might not be considered as ‘sex’ [8, 15, 34]. Increasing detection of oropharyngeal infections may partially explain the increase in gonorrhoea positivity. Literature shows a decreased susceptibility of gonorrhoea for ceftriaxone and cefotaxime among FSW in the Netherlands [35]. The threat of emerging resistance of gonorrhoea against the current first line treatment, in combination with the increasing gonorrhoea positivity rate among FSW raises concerns. Apart from condom advice for oral sex, FSW practicing oral sex should always be tested oropharyngeal to actively detect oropharyngeal infections present.

Female sex workers who were younger, previously diagnosed with an STI, or who were notified through partner notification, were at higher risk for chlamydia and gonorrhoea, which is in line with the literature [20, 36]. While East-European FSW were at higher risk for gonorrhoea compared with native Dutch FSW, they were at lower risk for chlamydia. We found no literature to support this. Perhaps this could be explained by the high chlamydia prevalence among heterosexual men and women in the Netherlands, and/or a higher prevalence of gonorrhoea among East-European FSW [12, 32].

The higher STI positivity rate among consultations at the clinic compared to consultations performed during outreach activities is notable. The Prostitution & Health Centre in Amsterdam, which provides care for commercial sex workers, reported comparable results in their annual report [37]. There are several possible explanations for the difference in STI positivity rate between STI clinic and outreach consultations. First, FSW with STI related symptoms or FSW who experienced condom failure actively visit the STI clinic for STI testing. Second, during outreach nurses will redirect FSW with symptoms to the STI clinic for STI testing and physical examination. Third, since only commercial sex workplaces with a permit are obligated to provide the STI clinic access, the outreach consultations will mainly be among legal FSW. Workplaces with a permit are stimulated by the public health services to promote a safe-sex policy and they often have guidelines for condom use and STI testing (personal communication with health care workers). We expect that FSW without a permit will mainly be tested at the STI clinic, since their workplaces are not visited by the STI clinic. We cannot exclude that higher STI rates would be found among these FSW.

Not all FSW (with or without a permit) will be tested at the STI clinic. Reaching FSW currently not seen by STI clinics and FSW outside workplaces with a permit remains a challenge for public health services. Some STI clinics already do internet searches to detect individuals who sell sex and some STI clinics try to visit workplaces without a permit where selling of sex is suspected in order to reach FSW who are currently not seen.

### Strengths and limitations

A strength of our study is that we used data from all STI clinics in the Netherlands, which means we covered the whole FSW population tested by STI clinics. Furthermore, many consultations were included in the database and a wide time span was analysed.

A limitation of this study is that most variables (like being a FSW, testing history and condom use) were self-reported which could have led to recall bias and social desirability bias. For example, women reporting not to have exchanged sex for money or other valuable goods are not categorised as sex workers and thus are not included in this study.

Furthermore, we only have information on consultations and we cannot identify repeated consultations for the same FSW. Therefore, odds ratios of determinants associated with STI occurrence could be over- or underestimated since some consultations referred to the same FSW.

Finally, we cannot extrapolate these results to all FSW in the Netherlands. This data only contains FSW tested by STI clinics, but FSW can also visit their general practitioner for STI testing, conduct a self-test, or visit a clinic in their country of origin. This data also cannot be extrapolated to FSW who are not tested at all. We would expect to find a higher STI positivity rate among these FSW, since they are probably not reached by preventive public health services. We suggest further research into STI prevalence among FSW who are not currently tested by STI clinics and how to reach them for counselling and primary prevention.

## Conclusions

The positivity rate of any STI among FSW remained stable, but underlying this was a decreasing trend in the chlamydia positivity rate and an increasing trend in the gonorrhoea positivity rate, suggesting a shift in STI risks among FSW over time. Because of the potential transmission of STI, especially gonorrhoea, syphilis and HIV, to the general population and because of the increasing gonorrhoea positivity rate, preventive counselling and increasing awareness of STI risks among FWS remains important as well as regular STI testing of FSW. Condom use during oral sex should be promoted since gonorrhoea was frequently diagnosed in the oropharynx and because of the potential spread of antimicrobial resistant gonococci.

## Additional file

**Additional file 1:** Univariable logistic regression for determinants of infectious syphilis, HIV and hepatitis B among female sex workers, 2006 to 2013. Additional file 1 shows the crude odds ratios for the association between determinants included in the study and the outcome of having infectious syphilis, HIV or hepatitis B. Results are presented for female sex workers who were tested by the STI clinic in the Netherlands in the study period 2006 to 2013.

## Abbreviations

AOR: adjusted odds ratio
CI: confidence interval
ELISA: enzyme-linked immunosorbent assay
FSW: female sex worker
FTA: fluorescent treponemal antibody
NAAT: nucleic acid amplified tests
OR: odds ratio
RIVM: National Institute of Public Health and the Environment
TPHA: *Treponema pallidum* haemagglutination
TPPA: *Treponema pallidum* particle agglutination
VDRL: venereal disease research laboratory
STI: sexually transmitted infections
