# Supplementary material for: Sexually transmitted infections among female sex workers tested at STI clinics in the Netherlands, 2006–2013
Source: Emerg Themes Epidemiol. 2015 Aug 28;12:12. doi: 10.1186/s12982-015-0034-7 (PMC4552148; doi:10.1186/s12982-015-0034-7)
Supplement: Supplementary file 1 — Additional file 1: Univariable logistic regression for determinants of infectious syphilis, HIV and hepatitis B among female sex workers, 2006 to 2013. Additional file 1 shows the crude odds ratios for the association between determinants included in the study and the outcome of having infectious syphilis, HIV or hepatitis B. Results are presented for female sex workers who were tested by the STI clinic in the Netherlands in the study period 2006 to 2013. [file 12982_2015_34_MOESM1_ESM.docx]

**Additional file 1: Univariable logistic regression for determinants of infectious syphilis, HIV and hepatitis B among female sex workers, 2006 to 2013.**

|  | **Infectious syphilis N = 35485*** | | **HIV**  **N = 34012*** | | **Hepatitis B**  **N = 11983*** | |
| --- | --- | --- | --- | --- | --- | --- |
|  | N positive | Crude OR (95%CI) | N positive | Crude OR (95%CI) | N positive | Crude OR (95%CI) |
| **Age** |  | 1.02 (0.99-1.05) |  | 0.99 (0.96-1.03) |  | **0.97 (0.95-0.99)** |
| **Consultation year** |  | **0.76 (0.68-0.85)** |  | 0.93 (0.81-1.06) |  | 0.93 (0.86-1.02) |
| **Ethnicity†** |  |  |  |  |  |  |
| Native Dutch | 11 | Reference | 9 | Reference | 6 | Reference |
| Turkish | 0 | NA | 0 | NA | 0 | NA |
| North African | 4 | **9.08 (2.88-28.6)** | 1 | 2.71 (0.34-21.49) | 0 | NA |
| Surinamese | 1 | 1.55 (0.20-12.04) | 1 | 1.85 (0.23-14.60) | 0 | NA |
| Dutch Antilleans | 2 | **6.97 (1.54-31.55)** | 0 | NA | 0 | NA |
| Eastern European | 19 | **2.49 (1.18-5.23)** | 4 | 0.63 (0.19-2.03) | 66 | **13.55 (5.87-31.29)** |
| Sub-Saharan African | 0 | NA | 16 | **28.47 (12.54-64.62)** | 15 | **22.08 (8.54-57.19)** |
| Mid-South-American | 11 | **3.70 (1.60-8.55)** | 7 | **2.83 (1.05-7.60)** | 9 | **4.33 (1.54-12.20)** |
| Other European | 12 | **3.57 (1.58-8.10)** | 4 | 1.53 (0.47-4.50) | 13 | **8.75 (3.32-23.06)** |
| Asian | 3 | 2.35 (0.66-8.44) | 2 | 1.87 (0.40-8.66) | 12 | **12.53 (4.69-33.51)** |
| Unknown | 0 | NA | 0 | NA | 1 | 5.26 (0.63-43.97) |
| Rest | 3 | **8.50 (2.36-30.57)** | 1 | 3.61 (0.46-28.56) | 0 | NA |
| **STI in last two years**** |  |  |  |  |  |  |
| No | 35 | Reference | 34 | Reference | 100 | Reference |
| Yes | 15 | **3.23 (1.76-5.92)** | 3 | 0.69 (0.21-2.25) | 8 | 0.84 (0.41-1.73) |
| Unknown | 16 | **2.97 (1.64-5.38)** | 8 | 1.62 (0.75-3.50) | 14 | 1.35 (0.77-2.37) |
| **Ever HIV tested** |  |  |  |  |  |  |
| No | 18 | Reference | 14 | Reference | 47 | Reference |
| Yes, positive | 0 | NA | 0 | NA | 1 | 5.30 (0.68-41.34) |
| Yes, negative | 39 | **0.38 (0.22-0.65)** | 25 | **0.31 (0.16-0.60)** | 68 | **0.61 (0.42-0.88)** |
| Unknown | 9 | 1.04 (0.46-2.39) | 6 | 1.04 (0.39-2.64) | 6 | 0.48 (0.20-1.12) |
| **Symptoms‡** |  |  |  |  |  |  |
| No | 27 | Reference | 33 | Reference | 76 | Reference |
| Yes | 13 | 1.69 (0.87-3.29) | 7 | 0.77 (0.34-1.73) | 38 | **1.61 (1.09-2.38)** |
| Unknown | 11 | **4.23 (2.10-8.54 )** | 3 | 1.02 (0.31-3.33 ) | 6 | 1.84 (0.80-4.26) |
| **Being notified by partner notification‡** |  |  |  |  |  |  |
| No | 41 | Reference | 40 | Reference | 110 | Reference |
| Yes | 1 | 1.26 (0.17-9.20) | 0 | NA | 2 | 0.80 (0.20-3.26) |
| Unknown | 9 | **2.54 (1.23-5.23 )** | 3 | 0.95 (0.29-3.07 ) | 8 | 2.01 (0.97-4.14) |
| **Degree of urbanisation** |  |  |  |  |  |  |
| Very high | 31 | Reference | 16 | Reference | 29 | Reference |
| High | 8 | 0.59 (0.27-1.30) | 5 | 0.70 (0.26-1.91) | 15 | 1.08 (0.58-2.02) |
| Medium | 4 | 0.56 (0.20-1.59) | 2 | 0.53 (0.12-2.29) | 7 | 1.0 (0.44-2.29) |
| Low | 5 | 1.32 (0.51-3.41) | 2 | 0.99 (0.23-4.30) | 3 | 0.87 (0.26-2.86) |
| Very low | 0 | NA | 1 | 0.57 (0.08-4.23) | 2 | 0.61 (0.15-2.58) |
| Unknown | 18 | **0.52 (0.29-0.93)** | 19 | 1.03 (0.53-2.01) | 66 | 1.42 (0.92-2.20) |
| **Chlamydia** | 5 | 1.07 (0.43-2.67) | 3 | 0.93 (0.29-3.00) | 14 | 1.28 (0.73-2.23) |
| **Gonorrhoea** | 1 | 0.59 (0.08-4.24) | 1 | 0.88 (0.12-6.43) | 4 | 1.03 (0.38-2.80) |
| **Condom use at last sexual contact by type of partner‡** | |  |  |  |  |  |
| No condom use steady partner | 2 | Reference | 1 | Reference | 16 | Reference |
| Yes condom use steady partner | 1 | 2.85 (0.26-31.51) |  | NA | 1 | 0.40 (0.05-3.02) |
| No condom use casual partner | 1 | 1.10 (0.10-12.13) | 4 | 8.87 (0.99-79.38) | 8 | 0.91 (0.39-2.14) |
| Yes condom use casual partner | 11 | 1.90 (0.42-8.59) | 8 | 2.78 (0.35-22.20) | 26 | 0.69 (0.37-1.29) |
| Unknown | 2 | 2.55 (0.56-18.09) | 3 | 7.66 (0.80-73.69) | 4 | 0.72 (0.24-2.18) |
| * Only consultations included where a clinical test was done for that specific STI.  † Ethnicity was based on self-defined ethnicity for 2006 to 2010 and on (parental) country of birth from 2011 to 2013.  ‡ Only the years for which this was reported were included in the analyses  ** Diagnosed with chlamydia, gonorrhoea or syphilis in the last two years.  In bold: OR is statistically significant (p<0.05)  STI: Sexually transmitted infections; NA: not applicable; OR: Odds Ratio; CI: confidence interval | | | | | | |
